# Open-Source Biomedical Image Analysis Models: A Meta-Analysis and Continuous Survey

**DOI:** 10.3389/fbinf.2022.912809

**Published:** 2022-07-05

**Authors:** Rui Li, Vaibhav Sharma, Subasini Thangamani, Artur Yakimovich

**Affiliations:** ^1^ Center for Advanced Systems Understanding (CASUS), Helmholtz-Zentrum Dresden-Rossendorf e. V. (HZDR), Görlitz, Germany; ^2^ Bladder Infection and Immunity Group (BIIG), Department of Renal Medicine, Division of Medicine, University College London, Royal Free Hospital Campus, London, United Kingdom; ^3^ Artificial Intelligence for Life Sciences CIC, Dorset, United Kingdom; ^4^ Roche Pharma International Informatics, Roche Diagnostics GmbH, Mannheim, Germany

**Keywords:** machine learing, deep learning, open source, bioimaging, image analysis, medical imaging

## Abstract

Open-source research software has proven indispensable in modern biomedical image analysis. A multitude of open-source platforms drive image analysis pipelines and help disseminate novel analytical approaches and algorithms. Recent advances in machine learning allow for unprecedented improvement in these approaches. However, these novel algorithms come with new requirements in order to remain open source. To understand how these requirements are met, we have collected 50 biomedical image analysis models and performed a meta-analysis of their respective papers, source code, dataset, and trained model parameters. We concluded that while there are many positive trends in openness, only a fraction of all publications makes all necessary elements available to the research community.

## Introduction

The source code of data analysis algorithms made freely available for possible redistribution and modification (i.e. open source) has been beyond any doubt driving the ongoing revolution in Data Science (DS), Machine Learning (ML), and Artificial Intelligence (AI) (Sonnenburg et al., 2007; Landset et al., 2015; Abadi et al., 2016; Paszke et al., 2019). Encouraging open collaboration, the open-source model of code redistribution allows researchers to build upon their peers’ work on a global scale fueling the rapid iterative improvement in the respective fields (Sonnenburg et al., 2007). Conversely, “closed-source” publications not only hamper the development of the field but also make it hard for the researchers to reproduce the results disseminated in the research articles. While *de jure* all published work resides in the public domain, reverse engineering of an advanced algorithm implementation may often take weeks or months, making such works hard to reproduce.

Needless to say, open source comes in a great variety of shapes and kinds. Remarkably, just making the source code of your research software available publicly or upon request does not *per se* make it open source. Usage and redistribution of any original creation, be it a research article or source code, lies within the legal boundaries of copyright laws, which differ significantly from country to country. Therefore, for example, publicly available code without an explicit attribution of a respective open-source license cannot be counted or treated as open source. Due to the sheer diversity, it may be difficult to judge which specific license is right for one’s project. Yet the choice of the license must always be dictated by the project and the intent of its authors. Consulting the licenses list approved by the Open Source Initiative is generally considered to be a good starting point.

The importance of open source software for computational biomedical image analysis has become self-evident in the past 3 decades. Packages like ImageJ/Fiji (Schindelin et al., 2012; Schneider et al., 2012), CellProfiler (Carpenter et al., 2006), KNIME (Tiwari and Sekhar, 2007), and Icy (de Chaumont et al., 2011) not only perform the bulk of quantification tasks in the wetlabs but also serve as platforms for distribution of modules containing cutting-edge algorithms. The ability to install and use these modules and algorithms by researchers from various fields *via* a point-and-click interface made it possible for the research groups without image analysis specialists to obtain a qualitatively new level of biomedical insights from their data. Yet, as we transition into the data-driven and representation learning paradigm of biomedical image analysis, the availability of datasets and trained model parameters becomes as important as the open-source code.

The ability to download training parameters may allow researchers to skip the initial model training and focus on gradual model improvement through a technique known as transfer learning (West et al., 2007; Pan and Yang, 2010). Transfer learning has proven effective in Computer Vision (Deng et al., 2009) and Natural Language Processing (Wolf et al., 2020) domains (further reviewed in (Yakimovich et al., 2021)). However, the complexity of sharing the trained parameters of a model differs significantly between ML algorithms. For example, while model parameters of a conventional ML algorithm like linear regression may be conveniently shared in the text of the article, this is impossible for DL models with millions of parameters. This, in turn, requires rethinking conventional approaches to ML/DL models sharing under an open-source license.

In this review, we collate ML models for biomedical image analysis recently published in the peer-reviewed literature and available as open-source. We describe open-source licenses used, code availability, data availability, biomedical and ML tasks, as well as the availability of model parameters. We make the collated collection of the open-source model available *via* a GitHub repository and call on the research community to contribute their models to it *via* pull requests. Furthermore, we provide descriptive statistics of our observations and discuss the pros and cons of the status quo in the field of biomedical image analysis as well as perspectives in the general DS context. Several efforts to create biomedical ML model repositories or so-called “zoos” (e.g. bioimage. io) and web-based task consolidators (Hollandi et al., 2020; Stringer et al., 2021) have been undertaken. Here, rather than proposing a competing effort, we propose a continuous survey of the field “as is”. We achieve this through collating metadata of published papers and their respective source code, data, and model parameters (also known as weights and checkpoints).

### Continuous Biomedical Image Analysis Model Survey

To understand the availability, reproducibility, and accessibility of published biomedical image analysis models we have collected a survey meta-dataset of 50 model articles and preprints published within the last 10 years. During our collection effort, we have prioritized publications with accompanying source code freely available online. In an attempt to minimize bias, we made sure that no individual medical imaging modality or biomedical task represents more than 25% of our dataset. Additionally, we have attempted to sample models published by both the biomedical community (e.g. Nature group journals), engineering community (IEEE group journals and conferences), as well as models published as preprints. For each publication we have noted the biomedical imaging modality, biomedical task (e.g. cancer), the open-source license used, reported model performance with respective metric, whether the model is dealing with the supervised task, whether the model parameters can be downloaded (as well as the respective link), links to code and dataset. Noteworthy, performance reporting is highly dependent on a dataset or benchmark. Therefore, to avoid confusion or bias we have recorded the best-reported performance for illustrative purposes only. Identical performance on a different dataset should not be expected. For the purpose of this review, we have split this meta-dataset into three tables according to the ML task of the models. The full dataset is available on GitHub (https://github.com/casus/bim). To ensure the completeness and correctness of this meta-dataset we invite the research community to contribute their additions and corrections to our survey meta-dataset.

First display table obtained from our meta-dataset contains 14 models aimed at biomedical image classification (Table 1). The most prevalent imaging modalities for this ML task are computed tomography (CT) and digital pathology—both highly clinically relevant modalities. We noted that most publications had an open-source license clearly defined in their repositories. The consensus between the choices of metric is rather low, making it difficult to compare one model to the other. Although most models had both source code and datasets available, only 4 out of 14 models had trained model parameters available for download.

**TABLE 1:** Biomedical Image Classification Models. Here, AUC is Area under curve, CT is computed tomography.

Imaging Modality	Biomed Task	License	Reported Performance	Parameters Download	References
CT	Lung tumor	Apache-2.0	0.93 Accuracy	No	LaLonde et al. (2020)
CT	Lung tumor	MIT	0.76 AUC	No	Guo et al. (2020)
CT	Pulmonary nodule	GPL-3.0	0.90 Accuracy	No	Zhu et al. (2018a)
CT	Pulmonary nodule	MIT	0.96 AUC	No	Al-Shabi et al. (2019)
CT	Pulmonary nodule	MIT	0.95 AUC	No	Dey et al. (2018)
Dermatoscopy	Skin tumor	N/a	0.93 Accuracy	No	Datta et al. (2021)
Dermatoscopy	Skin tumor	MIT	0.81 AUC	Yes	Zunair and Ben Hamza, (2020)
Mammography	Breast tumor	CC BY-NC-ND 4.0	0.93 AUC	Yes	Shen et al. (2021)
Digital Pathology	Breast tumor	CC BY-NC-ND 4.0	0.63 F1	No	Pati et al. (2022)
Mammography	Breast tumor	CC BY-NC-SA 4.0	0.84 Accuracy	Yes	Shen et al. (2019)
Digital Pathology	Breast tumor	MIT	0.93 Accuracy	Yes	Rakhlin et al. (2018)
Digital Pathology	Lung tumor	GPL-3.0	0.53 Kappa	No	Wei et al. (2019)
Digital Pathology	Lung tumor	MIT	0.97 AUC	No	Coudray et al. (2018)
Fluorescence microscopy	Host-pathogen interactions	N/a	0.92 Accuracy	No	Fisch et al. (2019)

The second display table contains 25 models (Table 2) aimed at biomedical image segmentation—a task relevant for obtaining quantitative insights from the biomedical images (e.g. size of the tumor). Similarly, to the models for biomedical image classification, the vast majority of the segmentation models have a well-defined open-source license with only a few exceptions. Again, similarly to the classification models, the consensus between performance metric choices is rather low, although Dice score reports clearly dominated. Conversely, the percentage of models with pre-trained parameters available for download is slightly higher than in the case of the classification models (36% vs 29%). However, over half of the models do not provide pre-trained parameters for the download for both segmentation and classification tasks.

**TABLE 2:** Biomedical Image Segmentation Models. Here, CT is computed tomography, DSC is Dice similarity coefficient, AP is Average Precision, IoU is Intersection over Union, DOF is Depth of field, AUC is Area under curve, SHG is Second harmonic generation microscopy.

Imaging Modality	Biomed Task	License	Reported Performance	Parameters Download	References
3D microscopy	Nuclei detection	MIT	0.937 AP	No	Hirsch and Kainmueller, (2020)
CT	Kidney tumor	GPL-3.0	0.95 Dice	No	Müller and Kramer, (2021)
CT	Pulmonary nodule	BSD-3-Clause	N/a	No	Hancock and Magnan, (2019)
CT	Pulmonary nodule	CC BY-NC-SA 4.0	0.55 IoU	Yes	Aresta et al. (2019)
CT	Pulmonary nodule	MIT	0.83 DSC	No	Keetha et al. (2020)
CT	Pancreas & Brain tumor	MIT	0.84 Dice	No	Oktay et al. (2018)
CT, Dermatoscopy	Lung tumor and Skin tumor	N/a	0.9965 Jaccard	No	Kaul et al. (2019)
CT	Brain tumor	Apache 2.0	0.89 Dice	No	Isensee et al. (2018)
MRI	Brain tumor	Apache 2.0	0.79 Dice	No	Wang et al. (2021)
MRI	Brain tumor	CC BY-NC-ND 4.0	0.76 Dice	No	Baek et al. (2019)
Digital Pathology	Breast tumor	CC BY-NC-ND 4.0	0.893 F1	Yes	Le et al. (2020)
Digital Pathology	Lung tumor	CC-BY	0.83 Accuracy	No	Tomita et al. (2019)
Digital Pathology	Multiple pathologies	MIT	N/a	No	Khened et al. (2021)
Electron microscopy	Multiple pathologies	MIT	0.5 VI	Yes	Lee et al. (2017)
Fluorescence microscopy	Cellular structures reconstruction	N/a	20 x Enhancement in DOF	Yes	Wu et al. (2019)
Fluorescence microscopy	Nuclei detection	BSD-3-Clause	0.94 Accuracy	Yes	Weigert et al. (2020)
Microscopy	Cellular reconstruction	N/a	0.69 AP	No	Hirsch et al. (2020)
MRI	Brain tumor	BSD-3-Clause	0.87 Dice	Yes	Wang et al. (2018)
MRI	Brain tumor	MIT	0.85 Dice	Yes	Havaei et al. (2017)
MRI	Brain tumor	MIT	0.90 Dice	No	Isensee et al. (2018)
MRI	Brain tumor	MIT	0.91 Dice	No	Myronenko, (2019)
SHG	Bone disease	GPL-3.0	0.78 Accuracy	No	Schmarje et al. (2019)
Time-lapse microscopy	Nuclei detection	N/a	0.92 Accuracy	Yes	Shailja et al. (2021)
Ultrasound imaging	Intraventricular hemorrhage	MIT	0.89 Dice	No	Valanarasu et al. (2020)
MRI	Brain tumor	N/a	0.81 Dice	Yes	Larrazabal et al. (2021)

Finally, we have also examined biomedical image analysis models aimed at less popular ML tasks including data generation, object detection or reconstruction (Table 3). Apart from digital pathology, CT scans this group of models also contains light and electron microscopy. Remarkably, only 19% of models in this group had downloadable model parameters. At the same time, almost all the models in this group had well attributed open-source licenses. This may suggest that parameter sharing is not very common in highly specialized fields like microscopy. Interestingly, for this and other groups of ML tasks, we have found that parameter sharing was more common in models submitted as a part of a data challenge. This may be simply a result of data challenge participation conditions.

**TABLE 3:** Other Biomedical Image Models. Here, CT is computed tomography.

Imaging Modality	Biomed Task	ML Task	License	Parameters Download	References
Mammography	Breast tumor	Classification & Detection	N/a	Yes	Ribli et al. (2018)
Fluorescence microscopy	Cellular structures reconstruction	Data generation	Apache-2.0	No	Eschweiler et al. (2021)
CT	Pulmonary nodule	Detection	Apache-2.0	No	Zhu et al. (2018b)
CT	Pulmonary nodule	Detection	MIT	No	Li and Fan, (2020)
Digital Pathology	Multiple pathologies	Graph embedding	AGPL 3.0	No	Jaume et al. (2021)
Mammography	Breast tumor	Image Inpainting & Data generation	CC BY-NC-ND 4.0	Yes	Wu et al. (2018)
Confocal microscopy	Cellular structures reconstruction	Reconstruction	Apache-2.0	No	Vizcaíno et al. (2021)
Cryo-electron microscopy	Cellular structures reconstruction	Reconstruction	GPL-3.0	No	Zhong et al. (2019)
Cryo-electron microscopy	Protein structures reconstruction	Reconstruction	GPL-3.0	No	Ullrich et al. (2019)
Electron microscopy	Cellular structures reconstruction	Reconstruction	N/a	No	Guay et al. (2021)
3D microscopy	Image acquisition	Reconstruction	BSD-3-Clause	No	Saha et al. (2020)

### Trends Meta-Analysis in Biomedical Image Analysis Model

**FIGURE 1:**
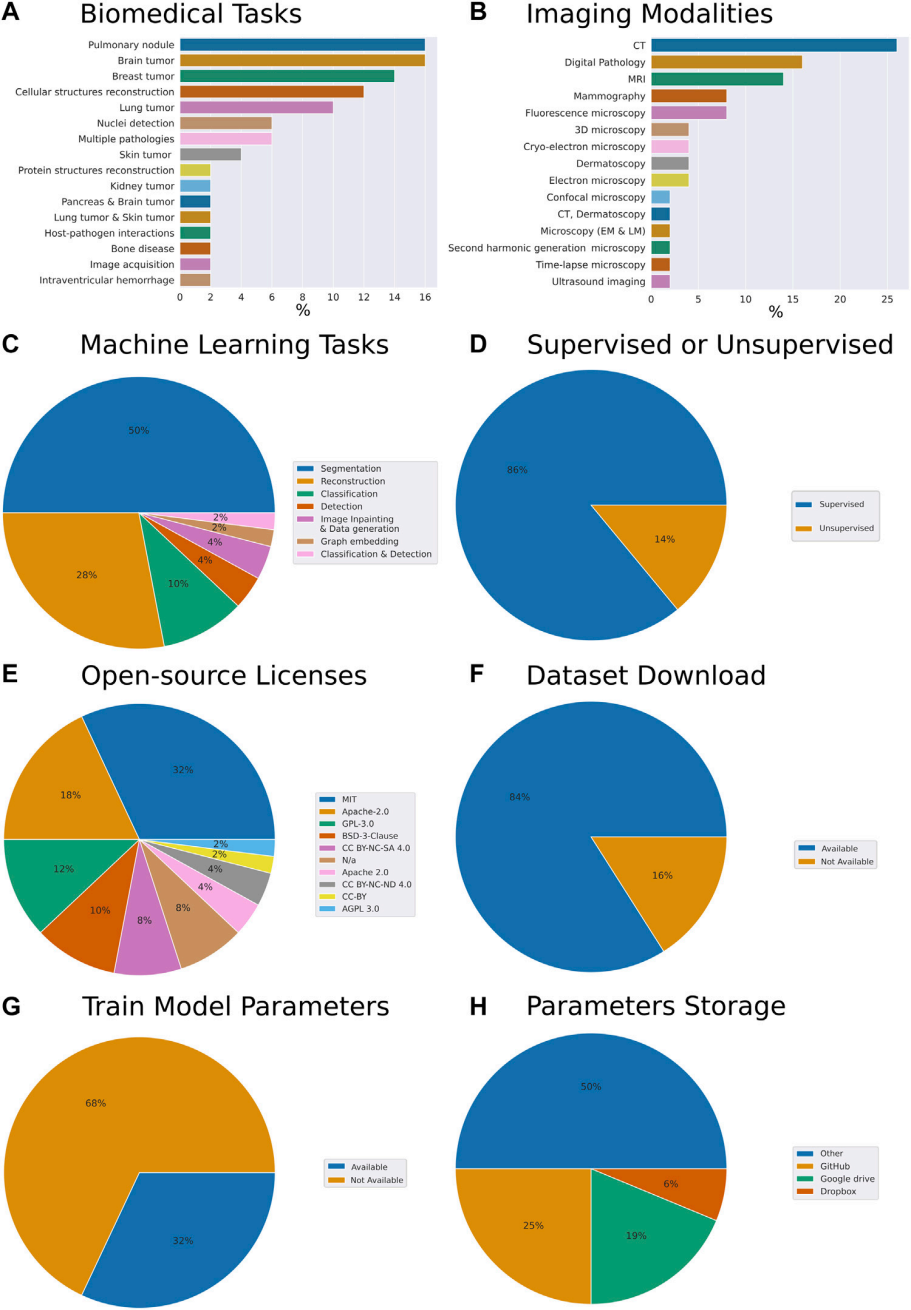
Meta-analysis of trends in open-source biomedical image analysis models **(A)** Biomedical tasks overview and breakdown in our collection **(B)** Variety of imaging modalities **(C)** Machine learning tasks the models are aimed at **(D)** Are the ML algorithms used for supervised or unsupervised learning tasks **(E)** Prevalence of open source licenses used **(F)** Availability of datasets **(G)** Availability of trained model parameters **(H)** Prevalence of platforms used for trained model parameters sharing. Here, CT is computed tomography, MRI is magnetic resonance imaging.

To understand general trends in the collection of our open-source models we have computed respective fractions of each descriptive category we have assigned to each work. The assignment was performed through careful analysis of the respective research article, code repository, dataset repository, and the availability of the trained model parameters (Figure 1). While admittedly 50 papers constitute a relatively small sample size, we have made the best reasonable effort to ensure the sampling was unbiased. Specifically, the set of models we have reviewed addresses the following biomedical tasks (from most to least frequent): pulmonary nodule, brain tumor, breast tumor, cellular structures reconstruction, lung tumor, cell nuclei detection, multiple pathologies, skin tumor, protein structures reconstruction, kidney tumor, pancreas and brain tumor, lung tumor and skin tumor, host-pathogen interactions, bone disease, image acquisition, intraventricular hemorrhage (Figure 1A).

From the perspective of imaging modalities, the models we reviewed span the following: computed tomography (CT), digital pathology, magnetic resonance imaging (MRI), mammography, fluorescence microscopy, 3D microscopy, cryo-electron microscopy, dermatoscopy, electron microscopy, confocal microscopy, CT and dermatoscopy, light and electron microscopy, second harmonic generation microscopy, time-lapse microscopy, ultrasound imaging (Figure 1B). From the perspective of ML tasks these models covered the following: segmentation, reconstruction classification, object detection, imagine inpainting and data generation, graph embedding, classification, and detection (Figure 1C). 86% of the models we have reviewed were addressing supervised tasks and 14% unsupervised tasks (Figure 1D).

Within our collection of open-source models, we have noted that 32% of the authors have selected the MIT license, 18% have selected Apache-2.0, 12%—GPL-3.0, 10%—BSD-3-Clause license, 8%—CC BY-NC-SA 4.0 license. Remarkably, another 8% have published their code without license attribution, arguably making it harder for the field to understand the freedom to operate with the code made available with the paper (Figure 1E). Within these papers, 84% of the authors made the dataset used to train the model available and clearly indicated within the paper or the code repository (Figure 1F). Overall, this amounted to the vast majority of the works which we have selected to have a clear open-source license designation, as well as a dataset available.

Remarkably, while providing the model’s source code, as well as, in most cases, the model’s dataset, an impressive 68% of the contributions we have reviewed did not provide trained model parameters (Figure 1G). Breaking down by the publishers or repositories, 43% and 31% of papers published by Nature group and Springer respectively provided model parameters. However, only 25% of IEEE papers and 14% of arXiv preprints provided parameters. Altogether, the low percentage of shared parameters are suggesting that the efforts to reproduce these papers came with the caveat of provisioning a hardware setup capable of wielding the computational load required by the respective model. In some cases that requiresaccess to the high-capacity computing. Furthermore, this way, instead of simply building upon the models trained, the efforts of the authors would have to be first reproduced. Needless to say, should any of the papers become seminal these high-performance computations would have to be repeated time and time again, possibly taking days of GPU computation.

Interestingly, of the authors who have chosen to make the trained parameters available to the readers around 25% have chosen to deposit the parameters on GitHub, while 19% and 6% have opted for Google drive and Dropbox services respectively. The rest deposited their parameters on the proprietary and other services (Figure 1H).

## Discussion

The advent of ML and specifically representation learning is opening a new horizon for biomedical image analysis. Yet, the success of these new advanced ML approaches brings about new requirements and standards to ensure quality and reproducibility (Hernandez-Boussard et al., 2020; Mongan et al., 2020; Norgeot et al., 2020; Heil et al., 2021; Laine et al., 2021). Several minimalistic quality standards applicable to the clinical setting have been proposed (Hernandez-Boussard et al., 2020; Mongan et al., 2020; Norgeot et al., 2020), and while coming from slightly different perspectives they demonstrate an overlap on essential topics like the dataset description, comparison to baseline and hyperparameters sharing. For example, CLAIM (Mongan et al., 2020) and MINIMAR (Hernandez-Boussard et al., 2020) approaches aim to adhere to a clinical tradition. Authors define a checklist including a structure of an academic biomedical paper, requiring either a lengthy biomedical problem description (CLAIM) or descriptive statistics of the dataset’s internal structure (MINIMAR). At the same time, MI-CLAIM (Norgeot et al., 2020) aims to adhere to the Data Science tradition, focusing specifically on data preprocessing and baseline comparison. Remarkably, even though item 24 of the CLAIM checklist explicitly mentions the importance of specifying the source of the starting weights (parameters) if transfer learning is employed, all three approaches fail to explicitly encourage sharing of the trained model parameters. Instead of proposing yet another checklist, the current survey aims to understand to extend to which the model parameters are shared in the biomedical image analysis field and emphasize the importance of parameters sharing to foster reproducibility in the field.

The past 3 decades have successfully demonstrated the viability of the open-source model for the research software in this field, as well as the role of open-source software in fostering scientific progress. However, the change of modeling paradigm to DL requires new checks and balances to ensure the results are reproducible and the efforts are not doubled. Furthermore, major computational efforts inevitably come with an environmental footprint (Strubell et al., 2020). Making parameters of the trained models available to the research community not only could minimize this footprint, but also open new prospects for the researcher wishing to fine-tune the pre-trained models to their task of choice. Such an approach proved incredibly fruitful in the field of natural language processing (Zhang et al., 2020).

Remarkably, in the current survey, we have found that only 32% of the biomedical models we have reviewed made the train model parameters available for download. On one hand, such a low number of trained models available for download may be explained by the fact that many journals and conferences do not require trained models to warrant publication. On another hand, with parameters of some models requiring hundreds of megabytes of storage, there are not many opportunities to share these files. Interestingly, while some researchers shared their trained model parameters *via* platforms like GitHub, Google drive, and Dropbox, the vast majority opted for often proprietary sites to share these parameters (Figure 1H). In our opinion, this indicates the necessity of hubs and platforms for sharing trained biomedical image analysis models.

It is worth noting that most cloud storage services like Google drive or Dropbox are more suited for instant file sharing rather than archival deposition of model parameters. These storage solutions don’t offer data immutability or digital object identifiers attached to them, and hence can simply be overwritten or disappear leaving crucial content inaccessible. Authors opting for self-hosting of model parameters also likely underestimate the workload of the long-term serving of archival data. Instead of the aforementioned approaches to model sharing, one should take advantage of efforts like BioImage.io, Tensorflow Hub (Paper, 2021), PyTorch Hub, DLHub (Chard et al., 2019), or similar in order to foster consistency and reproducibility of their results. Arguably, one of the most intuitive experiences of model parameters sharing for the end-users is currently offered by the HuggingFace platform in the domain of natural language processing. This has largely been possible through the platform’s own ML library allowing for improved compatibility (Wolf et al., 2020).

Interestingly, the vast majority of authors have chosen MIT and Apache-2.0 as their open-source licenses. Both Apache-2.0 and MIT are known for being permissive, rather than copyleft licenses. Furthermore, both licenses are very clearly formulated and easy to use. It is tempting to speculate that their popularity is a result of the simplicity and openness that these licenses offer.

However, noteworthy, our survey is limited to the papers we reviewed. To improve the representativeness of our meta-analysis, as well as encourage the dissemination of the open-source models in biomedical image analysis we call on our peers to contribute to our collection *via* the GitHub repository. Specifically, we invite the researchers to fork our repository, make additions to the content of the list following the contribution guidelines and merge them in *via* pull request. This way we hope to not only obtain an up-to-date state of the field but also ensure the code, datasets and trained model parameters are easier to find.
